# Versatile Biomaterial Platform Enriched with Graphene Oxide and Carbon Nanotubes for Multiple Tissue Engineering Applications

**DOI:** 10.3390/ijms20163868

**Published:** 2019-08-08

**Authors:** Simona-Rebeca Ignat, Andreea Daniela Lazăr, Aida Şelaru, Iuliana Samoilă, George Mihail Vlăsceanu, Mariana Ioniţă, Eugen Radu, Sorina Dinescu, Marieta Costache

**Affiliations:** 1 Department of Biochemistry and Molecular Biology, University of Bucharest, 050095 Bucharest, Romania; 2 Advanced Polymer Materials Group, University Politehnica of Bucharest, 011061 Bucharest, Romania; 3 Molecular Biology and Pathology Research Lab “MolImagex”, University Hospital Bucharest, 050098 Bucharest, Romania

**Keywords:** cellulose acetate, carbon nanotubes, graphene oxide, human adipose-derived stem cells, micro-CT

## Abstract

Carbon-based nanomaterials, such as graphene oxide (GO) or carbon nanotubes (CNTs) are currently used in various medical applications due to their positive influence on biocompatibility, adhesion, proliferation, and differentiation, as well as their contribution to modulating cell behavior in response to nanomaterial substrates. In this context, in this study, novel flexible membranes based on cellulose acetate (CA) enriched with CNT and GO in different percentages were tested for their versatility to be used as substrates for soft or hard tissue engineering (TE), namely, for their ability to support human adipose-derived stem cells (hASCs) adhesion during adipogenic or osteogenic differentiation. For this purpose, differentiation markers were assessed both at gene and protein levels, while histological staining was performed to show the evolution of the processes in response to CA-CNT-GO substrates. Micro-CT analysis indicated porous morphologies with open and interconnected voids. A slightly lower total porosity was obtained for the samples filled with the highest amount of GO and CNTs, but thicker walls, larger and more uniform pores were obtained, providing beneficial effects on cell behavior and increased mechanical stability. The addition of 1 wt% GO and CNT to the biocomposites enhanced hASCs adhesion and cytoskeleton formation. The evolution of both adipogenic and osteogenic differentiation processes was found to be augmented proportionally to the GO-CNT concentration. In conclusion, CA-CNT-GO biomaterials displayed good properties and versatility as platforms for cell differentiation with potential as future implantable materials in TE applications.

## 1. Introduction

Graphene (G), graphene oxide (GO), and carbon nanotube (CNT) are carbon-based nanomaterials with remarkable mechanical and physicochemical properties with the possibility of good biocompatibility. It is generally accepted that substrates coated with these nanomaterials are able to modulate the behavior of cells seeded on their surface due to the similar properties they offer in the natural extracellular matrix (ECM) [1,2,3,4,5].

Graphene (G), a typical two-dimensional (2D) material that consists of carbon atoms arranged in a honeycomb-like structure is known to affect a variety of stem cell behaviors including adhesion, spreading, growth, and differentiation [6]. Because of graphene’s highly hydrophobic character, more often its derivative, i.e., graphene oxide (GO), is preferred. Due to the presence of hydroxyl, epoxide, and carboxyl groups on the basal plane and edges of GO, covalent and noncovalent interactions can occur with proteins and other biomolecules [4,7].

Adipogenesis, osteogenesis, chondrogenesis, and neurogenesis are just some of the differentiation processes that were studied in the presence of graphene derivatives. The use of G and GO was found to be the most effective for adipogenesis and osteogenesis, facilitating both the absorption of differentiation factors and cell adhesion [8,9,10,11]. Lee et al. reported that G promotes osteogenesis in human mesenchymal stem cells (hMSCs), while GO has high affinity for insulin and facilitates adipogenesis [4]. Patel et al. reinforced this by reporting that a composite system of GO-polypeptide hydrogel (GO-P) enhances the expression of adipogenic biomarkers such as PPAR-γ, C/EBP-α, LPL, and AP2, as compared with a pure hydrogel system and a composite system of graphene incorporated hydrogel (G/P) [12]. Instead, Suhito et al. found that GO-coated substrates better promoted osteogenesis than adipogenesis of hASC [13].

In addition to GO, another carbon derivative has been investigated for tissue engineering purposes, namely, carbon nanotubes (CNTs). CNTs consist of a sp2 hybridized network of C atoms rolled to form single-walled (SWCNTs) or multi-walled CNTs (MWCNTs) that have exceptional physical, mechanical, and electrical properties [14,15,16]. Because of these properties, CNTs are incorporated into various biomaterials as nanofillers to improve the different properties or to introduce novel functionalities [17]. CNTs increase the surface roughness of the substrate, therefore promoting high adsorption of ECM proteins such as fibronectin and vitronectin [18]. In addition, CNTs provide electrical stimulation to the cells seeded on their surface and enhance the differentiation of hMSC towards cardiomyocyte lineage [19].

It has been shown that CNT-coated scaffolds are able to support and facilitate the proliferation of hMSCs and their lineage-specific differentiation, with most studies focusing on bone regeneration. Square-patterned and aligned CNT substrates have been reported to promote the expression of core binding factor alpha1, osteocalcin, and alkaline phosphatase [20,21,22,23]. To our knowledge, adipogenesis has not yet been studied using CNT-based substrates.

Aside from lineage-specific differentiation, CNTs are also capable of enhancing adhesion and neural differentiation of hMSCs cultured on their surface [24]. CNTs are used in tissue engineering research and they also represent the focus of other biomedical applications, such as controlled delivery of drugs or bioactive agents (vaccines, antibodies, proteins, and nanosensors, etc.), cell tracking, and labeling [25,26,27].

The combination of G and CNTs has been previously investigated. G-CNT coated hydroxyapatite polyetheretherketone (HAP/PEEK) scaffolds facilitate the attachment of hMSC, which display good viability, proliferation, and differentiation towards bone [28]. Oyefusi et al. reported that CNT-G coated hydroxyapatite (HA) substrates accelerated osteogenic differentiation of temperature-sensitive human fetal osteoblastic cell line (hFOB 1.19) as compared to CNT-G uncoated scaffolds [29].

Cellulose acetate (CA), perhaps the most commercially appealing ester derivative of cellulose, is characterized by fine molecular arrangement, low toxicity properties but poor structural stability, and mechanical strength [30]. CA can be cost-effectively processed into practical designs such as fibers, films, porous bulks, and most commonly for the synthesis of cost-effective asymmetric membranes [31]. CA’s long-established exercise in industry undermined its potential for advanced application and only recently surfaced as an auspicious material for tissue engineering substrates. Electrospun blends of CA were validated as remarkable wound dressing constituents with low-adherence and high healing support considering cell proliferation and collagen extracellular matrices deposition [32]. CA has been investigated for artificial blood vessels [33], skin grafts [34] and heart valve tissue engineering [31].

Phase inversion is a convenient and accessible method to precipitate polymers by submerging their casted liquid solutions in a nonsolvent bath. By constrained formulation to control perimeters and volumes, one can accomplish objects with precise size and shape control. Inherently, a phase separation technique facilitated by a solvent/nonsolvent exchange, phase inversion, caters to the need to fabricate porous items with desirable porosity [35]. The membrane polymorphism is probably most regulated by the demixing rates of the solute share which varies with the nature of the solvent, precursor concentration, temperature of the nonsolvent solution, and possible fillers/additives. The outturn of the coagulation process is frequently linked to the thermodynamics and kinetics that the additives render to the composite system [36]. CNTs, for instance, lower the permeability factors of polymer composites and limit the mutual diffusivities of both solvents and nonsolvents resulting in a more compact membranary template at equilibria [37]. The effect of GO on the kinetics of segregation has been outlined as an antagonistic contingent on the nature of the nonsolvent since it could both accelerate or delay it. The exchange process, when aqueous nonsolvents are involved, can be lagged due to the interactions beginning between the hydrophilic moieties of GO and water [38].

As a population of multipotent stem cells, hMSCs are able to differentiate into osteoblasts, chondroblasts, adipocytes, and tenocytes. They can even develop into skeletal muscle, smooth muscle, and cardiomyocytes. Furthermore, it has been shown that they have the potential to differentiate into neuron-like cells, hepatocytes, pancreatic cells, endothelial, epithelial or hematopoietic supporting cells [39]. Out of hMSC, human adipose-derived stem cells (hASCs) are a desirable source of cells for tissue engineering, mainly because they can be easily isolated in large quantities from lipoaspirate through minimal invasive procedures [40].

In a previous investigation [41], CA membranes strengthened with GO and CNTs outstood others with an impressive gain in mechanical attributes. Atomic force microscopy (AFM) mapping along the membrane surfaces depicted Young’s modulus distributions preferentially rising up from an average of 1.23 GPa to 251 GPa. The superiority of the carbon nanomaterial reinforced membranes was also confirmed by structural investigations involving Raman spectroscopy and FT-IR which advocated for the ordered structuration of the composite as a result of the nonbond interactions between CA and CNTs and GO distributed within. The novel composites featured outstanding compositional constancy, strong GO and CNTs embedded within the CA matrix, and excellent biological propriety in vitro [34]. In this context, the purpose of our study was to investigate the capacity and outturn of newly developed CA-CNT-GO membranes to support tissue engineering applications directed towards bone or soft tissues by examining the effect of different concentrations of GO and CNTs on hASCs during adipogenesis and osteogenesis.

## 2. Results

### 2.1. Micro-CT Analysis of CA/CNT/GO Systems

**Figure 1:**
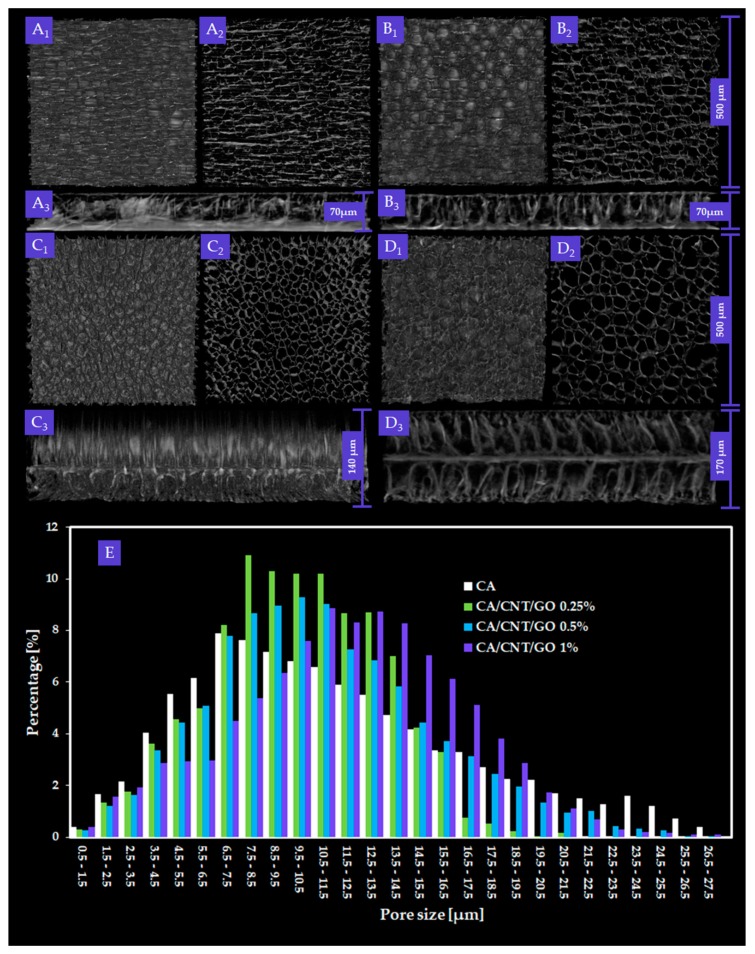
Three-dimensional (3D) renderings of: cellulose acetate (CA) (**A1**) top view, (**A2**) transversal section, and (**A3**) cross-section; CA, carbon nanotubes, and graphene oxide (CA-CNT-GO) 0.25% (**B1**) top view, (**B2**) transversal section, and (**B3**) cross-section; CA-CNT-GO 0.5% (**C1**) top view, (**C2**) transversal section, and (**C3**) cross-section; and CA-CNT-GO 1% (**D1**) top view, (**D2**) transversal section, and (**D3**) cross-section; with measured pore size distribution (**E**). Overall, for images A1–2, B1–2, and C1–2, scale bar is 500 µm. Widths of membrane cross-section are scaled particularly.

Figure 1 depicts the CTVox three-dimensional (3D) rendering of CA, CA-CNT-GO 0.25 wt%, CA-CNT-GO 0.5 wt%, and CA-CNT-GO 1 wt% two-dimensional (2D) slices depicting the membranes dominant morphology.

Wall and pore measurements were completed after a classic two-step image processing technique. Reconstructed tomograms are monochromes consisting of spectra of grey shades pixels spanning from white to black. First, thresholding is a binary filter which parts the greyscale spectra in two with a limit grey tone value. The thresholding value (9 to 255 greyscale) is manually selected by the microCT operator and stands for the boundary tone separating the solid matter from the environment. After thresholding, the filtered images consisted of only black and white, without intermediary grey tones. White depicts the membrane itself, while the empty space (pores and environment) is depicted in black. Despeckling is a technique suitable for the removal of scanning residue from the dataset; more accurately, by despeckling, the white pixels extraneous to the sample are removed allowing the execution of further accurate measurements. Structure thickness is a term used to describe the width of the sample walls as a function of the product between the number of white pixels and the scanning resolution, referred to as “pixel size”. Conversely, structure separation depicts the pore distribution based on the number of black pores and image pixel size. The metric size of the sample is translated in pixels through pixel size parameter.

**Figure 2:**
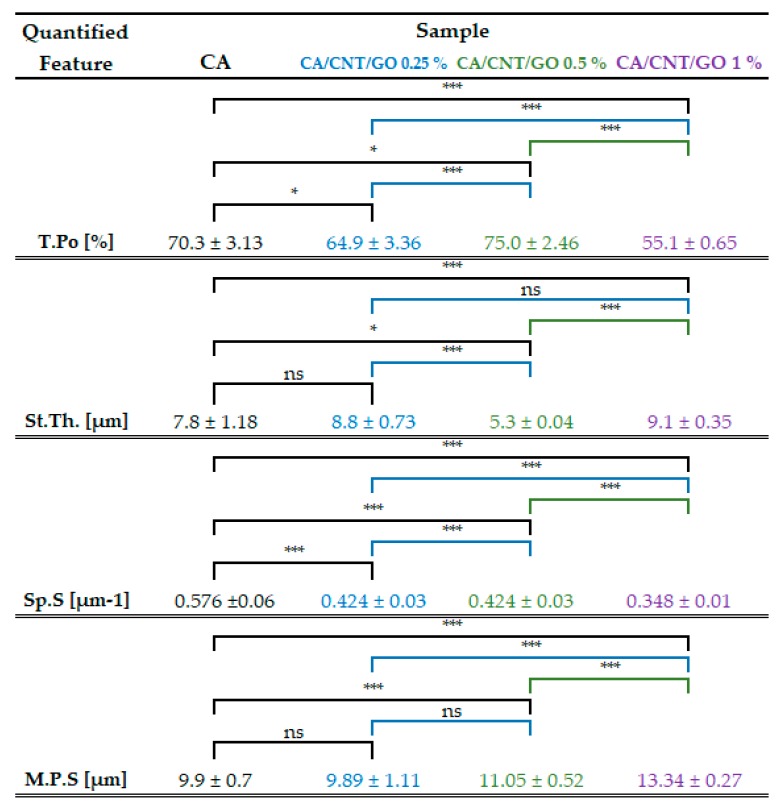
Tabulation of the calculated values for total porosity (T.Po), structure thickness (St.Th), and specific surface (Sp.S) appraised as the ratio of object surface and object volume and mean pore size (M.P.S.). The values were averaged after 3D analysis carried out in CTAn for 4 distinct proportionate volumes of interest (VOIs) per composite sample. Standard deviation and statistical significances are illustrated related to the CA control and each CNT and GO addition. (* *p* < 0.05; *** *p* < 0.001; ^ns^ *p* > 0.05).

By micro-CT analysis, overall, interconnected pore networks were featured in all specimens, with an important degree of isotropy along the longitudinal membrane axis. Closed pores, amounting to less than 0.1% of the absolute sample volume, were present in the samples. In Figure 2, total porosity, structure thickness, and specific surface are compiled. Specific surface was explicit as the ratio between object surface and object volume, which dictates a strong dependence with the total porosity. With low GO-CNT filling (0.25 wt%), the values for CA porosity and specific surface decrease, while at 0.5 wt%, total porosity (T.Po) and structure thickness (St.Th) reach maximum values. Contrarily, upon 1 wt% GO-CNT enrichment, T.Po and St.Th of the CA membrane reach minimum values as a result of the compactness variations (structure thickness) of the composite. In CTAn, St.Th is the weighted average measure of the material walls within the sample. There are important variations in St.Th occurring with GO-CNT supplementation in the CA matrix. The St.Th changes in reverse with total porosity for all samples which is in complete agreement with prior reports on CA composite membranes [41].

Pore size distribution of the composite membranes and the control were calculated using CTAn Bruker software for each of the four volumes of interest (VOIs) constrained per sample The ratios of pore size values were automatically provided in fixed size domains, i.e., image pixel size value and three times image pixel size. For this reason, the smallest pore feature that could be identified in the datasets was equal to the image pixel size (500 nm). The data depicted in the histogram (Figure 1E) are the weighted average of the values obtained from the VOIs of each material. A progressive increase of T.Po in the membranes with GO and CNTs was not found, however, an unequivocally linear trend arises in pore augmentation, as well as membrane thickness. In order to determine the mean pore size (M.P.S.), the weighted averages of the mean interval values in the VOIs were calculated, for each CA formulation. The M.P.S. is similar for CA-CNT-GO 0.25% and the control (less than 10 µm) and increases with GO and CNTs content to 11.05 and 13.34 µm, respectively. In cross-section, the measured membrane thickness was 70 µm for CA and CA-CNT-GO 0.25%, whereas, in the cases, of CA-CNT-GO 0.5% and CA-CNT-GO 1%, thicknesses increased to 140 µm and 170 µm.

Both pore size distribution and pore shape were altered as a result of the addition of GO and CNTs. This could be attributed to the mixture’s gradual increase in hydrophobicity upon the addition of GO and CNTs. Hence, the affinity of the casting composite solution mixture to the nonsolvent weakens. Upon nonsolvent immersion, the hydrophobic filler starts to segregate towards the membrane edges and initiates a premature demixing surge that also engages the CA network. The tendency to generate larger pores within the membrane is in direct correlation with the nanofiller content. We assume that 0.5 wt% GO-CNT concentration is above a threshold value, whereby the repulsion kinetics of the hydrophobic nanomaterial and nonsolvent could generate enough energy to engage large volumes of the polymer. After the contact with water, CNTs and GO (when in a higher amount 0.5 and 1 wt%) start to thrust from within the matrix and congest the medial domain bilaterally, generating an additional partition through the medial lamina. This phenomenon is facilitated by a large interfacial contact area between GO and CNTs with the enveloping polymer, on the grounds of the immense surface area of the nanomaterial and the excellent dispersion efficiency.

The process of phase inversion occurs along with the morphological changes of the polymer template. Additionally, the nonpolar GO-CNT mixture, upon interacting with the polar nonsolvent, is kinetically stimulated to migrate outward from the CA matrix. Therefore, broader pores are engendered as a result of a co-stimulation of the developing membrane walls and repulsion-mediated motility of the GO-CNT phase in the framework induces further strain from within walls, which adds to the solvent replacement porogenic process.

### 2.2. Cell Adhesion of hASCs on CA-CNT-GO Films

**Figure 3:**
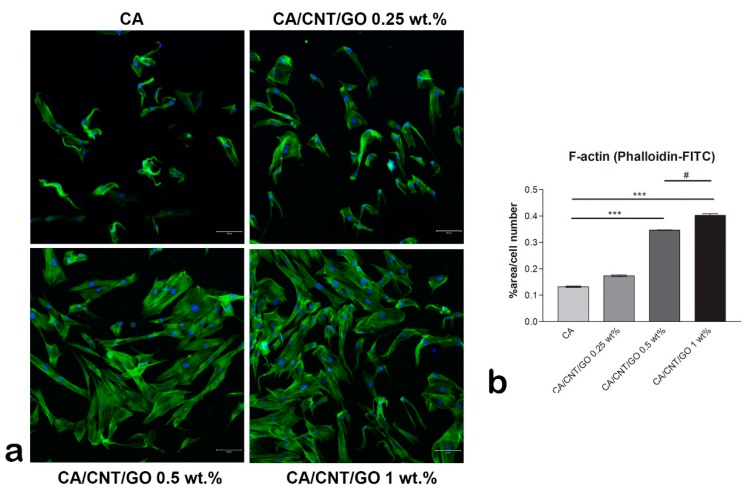
Cytoskeleton developed by human adipose-derived stem cells (hASCs) cultivated in contact with CA-CNT-GO materials, scale bar 100µm. (**a**) Fluorescence images of the actin filaments (green) and nuclei (blue) of hASCs and (**b**) quantification of the phalloidin-FITC staining normalized to cell number. Statistical significance: ^#^ *p* < 0.05; *** *p* < 0.001.

Immunofluorescence staining of actin filaments showed the cells’ cytoskeleton developed in contact with the materials (Figure 3). On the CA membrane, the actin remained condensed and fewer cells adhered as compared to the GO-CNT enriched films. As the GO and CNT content increased, the actin developed in filaments, corresponding to a spindle-like shape. This suggests the positive influence of CNTs and GO on the cellular adhesion. Moreover, the GO and CNT content influenced the number of cells that adhered to the substrates, as there are significantly more cells on the 1 wt% GO-CNT enriched material than on the films with lower concentrations.

### 2.3. Multiple Differentiation of hASCs on CA-CNT-GO Films

In this study we used CA as a control for showing multiple differentiation of hASCs on CA-CNT-GO films. We evaluated the ability of CA-CNT-GO films to promote the differentiation of hASCs into specific tissue lineages such as osteogenic and adipogenic.

#### 2.3.1. Adipogenic Differentiation Confirmation at the Histological Level

**Figure 4:**
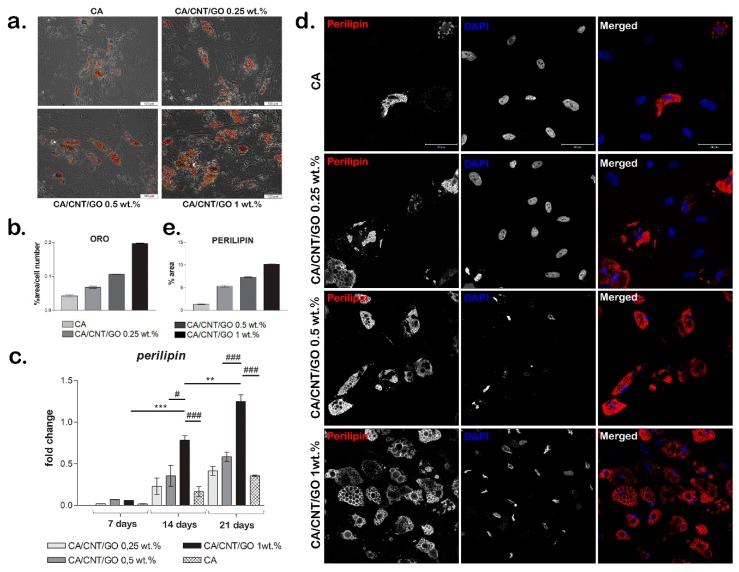
Evaluation of hASCs adipogenic differentiation on CA-CNT-GO materials: (**a**) Oil Red O staining for lipid droplets, scale bar 100 µm; (**b**) quantification of the Oil Red O staining normalized to cell number; (**c**) perilipin expression; Statistical significance: ^#^ *p* < 0.05; ** *p* < 0.01; ***^/###^ *p* < 0.001; (**d**) immunofluorescence for adipogenic marker perilipin. scale bar 50 µm; and (**e**) quantification of the immunofluorescence staining of perilipin.

The ability of cells to undergo adipogenesis on the materials was evaluated with Oil Red O staining. We observed a higher accumulation of intracellular lipids in hASCs grown on the CA-CNT-GO biomaterials than those cultivated on CA at seven and 21 days. The cells had large Oil Red O-positive lipid droplets within their cytoplasm (Figure 4a,b). At seven days, a low amount of intracellular lipids was detected on CA-CNT-GO 0.25%, while at 21 days the lipid droplets could be better observed, they were present in greater numbers and larger volume than the ones on CA. CA-CNT-GO 0.5% stimulated adipogenic differentiation better than the control or CA-CNT-GO 0.25%, although the changes in lipid amount were not as well observed at seven days as compared to 21 days, when more lipid droplets could be found. On the basis of the results, the material that best promoted adipogenic differentiation was CA-CNT-GO 1%. The intracellular lipids were already well defined at seven days as compared with the control and the other concentrations of GO-CNT films, CA-CNT-GO 1% had the most well-defined lipid droplets. These results confirm perilipin gene and protein expression levels and the evolution of the adipogenic differentiation process in our systems.

#### 2.3.2. Gene and Protein Expression of Adipogenic Markers

Perilipin gene expression was assessed during 21 days of the experiment as a measure of the degree of cell differentiation when cultured in contact with CA-CNT-GO systems (Figure 4c). An overall profile showed higher perilipin levels in the presence of CNTs and GO in the material than in the CA reference.

At seven days, no significant changes were observed, while at 14 days for CA-CNT-GO 1 wt% the level of perilipin expression was significantly higher than that observed for CA-CNT-GO 0.5 wt% (*p* < 0.05) and CA (*p* < 0.001). Likewise, after 21 days of differentiation, there was a significant change in the level of perilipin for CA-CNT-GO 1% as compared with the one registered for the control (*p* < 0.001) and CA-CNT-GO 0.5 wt% (*p* < 0.001).

We also observed statistically significant changes in gene expression when comparing between the same material at different times. There was a significant change (*p* < 0.05) in the level of perilipin expression for CA-CNT-GO 0.25 wt% at 21 days as compared to the gene expression registered for the same biomaterial at seven days. A similar result was obtained for CA-CNT-GO 0.5 wt%, with a higher perilipin expression (*p* < 0.01) at 21 days as compared to seven days. For CA-CNT-GO 1 wt% the rise in gene expression was statistically significant with *p* < 0.001 when comparing the expression registered for the film at 14 days and seven days, with *p* < 0.01 for the change in the level of perilipin registered at 21 days as compared to 14 days and *p* < 0.001 for the high expression of perilipin at 21 days in relation to the expression for CA-CNT-GO 1 wt% at seven days.

The expression of perilipin was detected on all four biomaterials and the change in its level from seven to 21 days is clearly noticeable. These findings suggest that CA-CNT-GO films better promote the process of adipogenic differentiation as compared with the CA control.

Perilipin gene expression results were also confirmed at the protein level by fluorescence microscopy after 21 days of adipogenic differentiation. We observed that perilipin was clearly better expressed for the CA-CNT-GO biomaterials as compared with the neat CA control (Figure 4d,e). Even a small concentration (0.25 wt%) of GO and CNTs favored the accumulation of lipids in the differentiating cells and an increase of perilipin expression as compared with the control, enhancing both the positive effect along with the concentration. The highest perilipin accumulation covering lipid droplets was detected for CA-CNT-GO 1 wt%.

#### 2.3.3. Osteogenesis Confirmation at the Histological Level

**Figure 5:**
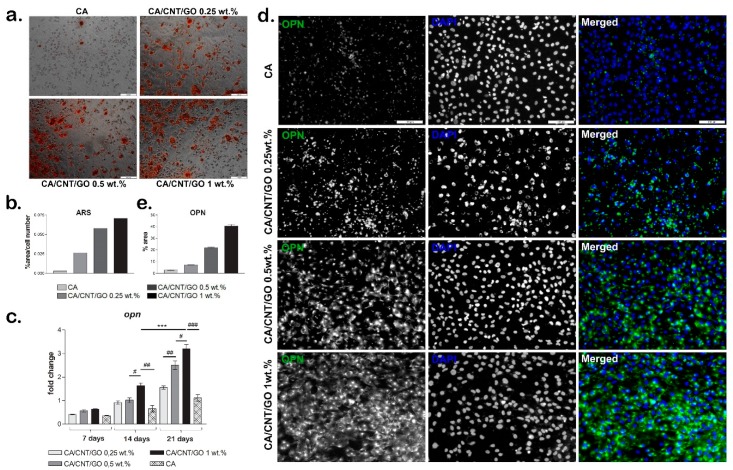
Evaluation of hASC osteogenic differentiation on CA-CNT-GO materials: (**a**) Alizarin Red S staining for measuring the level of mineralization, scale bar 100 µm. (**b**) quantification of the Alizarin Red S staining normalized to cell number, (**c**) osteopontin expression, Statistical significance: ^#^ *p* < 0.05; ^##^ *p* < 0.01; ***^/###^ *p* < 0.001, (**d**) immunofluorescence for osteogenic marker osteopontin (*OPN*), scale bar 100 µm and (**e**) quantification of the immunofluorescence staining of *OPN*.

The capacity of hASCs to undergo osteogenesis when cultivated in contact with CA-CNT-GO films was evaluated by Alizarin Red S staining (Figure 5a,b). The results showed overall that osteogenesis was developed more efficiently on CA-CNT-GO films than in the control. On CA, the cells became round, a sign that osteogenesis was induced, but only a few small mineralization nodules were detected. The progress of the osteogenic differentiation was limited on CA even after 21 days of differentiation as compared to the level of differentiation on the CA-CNT-GO films. As the concentration of GO and CNTs increased, the level of mineralization was improved as well, with the greatest level of mineralization observed on CA-CNT-GO 1 wt% film.

#### 2.3.4. Gene and Protein Expression of Osteogenic Markers

The gene expression of the osteogenic marker, osteopontin (*OPN*), was evaluated by qPCR at 7, 14, and 21 days of osteogenic differentiation (Figure 5c). Overall, the gene expression levels of *OPN* were found to be increased over the 21 days of osteogenesis, with lower levels for CA material as compared with the CA-CNT-GO materials. After seven days of osteogenic differentiation, the *OPN* levels were higher for the CA-CNT-GO materials as compared with the CA control, but with no statistically significant differences between them.

On the CA-CNT-GO 0.25 wt% and 0.5 wt% films, the expression level of *OPN* was similar after 14 days and higher than the *OPN* level for the control, but no statistically significant differences were registered between them. This suggests a positive effect on osteogenesis of CNTs and GO addition in the material, even when added in lower concentrations. However, the highest *OPN* level was registered for the 1 wt% GO-CNT-enriched material, with statistically significant differences as compared with the CA control and both CA-CNT-GO 0.25 wt% and 0.5 wt% materials. Compared to the control, the *OPN* level for CA-CNT-GO 1% was doubled, suggesting that GO and CNTs have a positive influence on the osteogenic differentiation in higher concentrations.

After 21 days of osteogenic differentiation, the lowest level of *OPN* was detected for the CA control, similar to the CA-CNT-GO 0.5 wt% *OPN* level from 14 days. This suggests that osteogenesis was induced in contact with CA as well, but at a much slower rate than on the CNT- and GO-enriched materials. As the GO-CNT concentration increased in the composition of the materials, the osteogenesis development was improved as well. The addition of even a low concentration of 0.25 wt% GO-CNT induced a higher *OPN* level as compared with the control. The addition of 0.5 wt% GO-CNT induced a statistically significant higher *OPN* level compared to CA-CNT-GO 0.25 wt% and CA reference. The highest *OPN* level, and therefore the most advanced osteogenic differentiation, was registered for CA-CNT-GO 1 wt%, with a statistically significant difference as compared with all the other material compositions.

The results of *OPN* gene expression evaluation were confirmed by the protein levels as well. After 21 days of osteogenic differentiation, we evaluated the protein level of osteogenic marker osteopontin in fluorescence microscopy (Figure 5d,e). On the control CA, *OPN* was only poorly expressed as comparing with its expression on the CA-CNT-GO films. Even the smallest concentration of GO-CNT 0.25 wt% clearly enhanced *OPN* expression as compared with the CA. Along with the increase of the GO-CNT concentration from 0.25 wt% to 1 wt%, an increasing *OPN* expression profile was obtained, suggesting the positive effect of GO and CNTs on the osteogenic differentiation process. The highest level of *OPN* was observed on the CA-CNT-GO 1 wt% material.

According to these findings, CA-CNT-GO films could be used for stem cell and tissue engineering applications because they have great potential to enhance hASCs’ differentiation selectively.

## 3. Discussion

In this study, we evaluated the potential of a CA membrane enriched with CNTs and GO in different concentrations for tissue engineering applications. The biocompatibility of the materials was previously assessed and confirmed in one of our studies [41]. On the basis of the positive biocompatibility results of CA-CNT-GO materials, this study investigated their properties and also their potential to support cell adhesion and to promote hASCs osteogenic and adipogenic differentiation processes to prove their versatility. We chose to use mesenchymal stem cells (hASCs) based on their potential to differentiate to multiple lineages such as osteogenic, adipogenic, and chondrogenic, etc. which allowed us to test cell response to the same substrate biomaterial, but in the context of different signaling triggered by either adipogenic or osteogenic inducers.

With regards to the morpho-structural analysis of CA-CNT-GO materials by micro-CT, it could be argued that GO and CNTs decreased T.Po and Sp.S while incrementing St.Th by means of a pore patterning mechanism. Furthermore, carbon nanomaterial addition remodeled the interior wall network of the membranes by increasing its degree of compactness. Figure 1, sets A and B expose the superficial features (A1, B1), internal porosity (A2, B2), and cross-sectional layout (A3, B3) of the control and CA-CNT-GO 0.25 wt%. The membrane surface is rather smooth, with creases along the edges of the circular pores beneath the surface. In cross-section, two parallel exterior facades imbedded by a dense network of pillar-like short walls are exposed, revealing a sandwich-like morphology. Peculiarly, upon increasing the GO-CNT addition (≥0.5 wt%), the pore domain is split by a novel component, a midway horizontal wall parallel to the peripheral surfaces. In Figure 1, set C3, one of the exterior walls was probably too thin (<500 nm) and thus attenuated during the scan, however, different morphologies can be observed on the two sides of the center lamina, given by the different thickness, density, and orientation of the anchoring walls. By comparison, in set C4, where the GO-CNT content is maximal, such distinctions are undermined due to the more compact nature of the sample.

Compactness degree is a direct implication of the GO-CNT suspension admixing with the initial CA scaffolding. From a rheological point of view, the carbon-based filling evenly integrated within CA solution volume induces a certain degree of heterogeneity. For instance, low amounts of GO are known to lower the dispersant viscosity, while CNTs can thicken the polymer template [42]. As a result, possible kinetic fluctuations can appear at a molecular scale; it is the authors opinion that thermodynamic balance is reached postliminary after CA coagulation [43]. Solvent removal equilibrates the systems, however, the highly energetic process itself occurs distinctively for each composite formulation. As addressed before, the porogenesis specificity infers from the filler share in the CA matrix. In a synergistic proceeding with solvent replacement, polarity discordances set up between nonsolvent and GO-CNT doublet fuel the nanoparticles preferential effusion from within the matrix. The attempted self-extraction of CNTs and GO is impeded by the strong lodging amidst the CA network. Nevertheless, during the course of coagulation, nonpolar carbon nanoparticles biasedly form molecular assemblages, engaging along the proximal CA concatenation. These arrays echo in the final membrane morphology. The additional energy in the unsettled thermodynamic systems is proportional with the filler amount and during its inflowing which is a consequences of its displacement mirror onto the surface tension of solidifying walls from the very beginning of porogenesis, through its quasi-states, prior to full pore template development. Consequently, the composites extent of ordering heightens with the CNTs and GO content through a pore shaping/wall densification mechanism. The presence of a centered third wall serves as additional mechanical support during the interaction with the cellular component. In agreement with the cell culture tests, the circular apertures within CA composites, similar to natural bone frameworks and medial lamina provide better durotactic gradients for cell attachment and differentiation, as compared with CA and CA-CNT-GO 0.25 wt%.

Highly porous structures, such as CA membranes, show better mechanical stability when medial walls form [41]. These kind of structures act like a scaffold providing support and better mechanical stress transfer. In this respect, it is the authors opinion that the walls of the pores act like a mechanical stress conductive media, whereas, the internal compact wall serves as a focusing center of force accumulation. In the case of cells with affinity to reliable substrates, this kind of environment is a durotaxis watershed for attachment and proliferation. Durotaxis, i.e., cell migration in accordance to committed gradient strength signals, is often correlated with cell velocity rendering the time frame for the scaffold colonization overall. The hASCs and hMSCs reciprocation to the extracellular stiffness is biased by myriads of mechanobiological cues particular per lineage, spanning the lengths and particularities of the simulated physiological landscape [44]. In our opinion, mechanotransduction is not the sole incentive of stem cell differentiation. All things considered, CA composites could be credited as bioinspired structures featuring a variety of inter-complementary assets sparking adipo- and osteogenesis in situ like dimensionally fit porosity, pore interconnectivity, and CNTs and GO load encouraging progenitors to sway to differentiation pathways.

The pore size distributions in the control and the three composite membranes follow a Gaussian right-skewed curve with a predilection to form a classical bell curve as the GO and CNTs content increases. Hence, as the matrix is more and more enriched with carbon nanomaterials and an increase in the share of larger pores arises. The widths of most pores (70% in the case of CA-GO-CNT, 88.2% for CA-GO-CNT 0.25%, 85.87% for CA-GO-CNT 0.5% and 88.7% in the case of CA-GO-CNT 1%, respectively) are encompassed between 5 and 20 µm. The share of pores with the size of less than 5 µm decreases from approximately 20% in the control to 12 in CA-GO-CNT 1%, favoring the genesis of pores with bigger volume. This propensity progresses preferentially in the porogenesis of 5–20 µm sized pores. In addition, the histogram outlines a predisposition of pore size evenness with GO and CNTs load. Above 20 µm, the pore share varies insignificantly and nonlinearly. The manner pore size increase is in agreement with acknowledged patterning of cell-friendlier architectures and consistent with hASC differentiation and gene expression data reported in this study, in spite of the overall diminution of the overall porosity value.

The synthesis of these biomaterials was closely correlated to their applicability. Therefore, each component was carefully chosen based on their specific properties. G was proven to be biocompatible and to have a positive influence on cellular adhesion and proliferation. As a graphene’s derivative, graphene oxide (GO) better interacts with proteins due to the presence of epoxide, carboxyl, and hydroxyl groups on the basal plane and edges. GO can better adsorb the proteins present in the serum, thus, providing more adhesion molecules involved in cell attachment [4].

In contact to GO-based materials, mesenchymal stem cells acquire an elongated shape and form focal adhesion points [45]. The addition of 3 wt% GO in a polysulfone-based material induced better developed actin filaments formation in murine preosteoblasts than the addition of lower concentrations [46]. The same trend was observed in our study as well, with better adhesion results for the materials with a higher concentration of GO and CNTs.

Carbon nanotubes (CNTs) represent another carbon-based nanomaterial that show promising potential for regenerative medicine due to their mechanical strength, flexibility, and electrical conductivity. CNT has been proposed as a new biocompatible substrate for cellular cultures. It has the ability to bind some of the proteins of the extracellular matrix (ECM), such as fibronectin, therefore, acting upon cellular behavior. The potential of CNTs to improve cell attachment and cell distribution was confirmed by many studies. On the basis of the results of a study conducted by Sheikholeslam et al. [47], it was concluded that the addition of CNTs in the composition of a hydrogel promoted better cell adhesion and more equally spread cells than in the hydrogels without CNTs. In another study by Das et al. [48], canine mesenchymal stem cells (cMSCs) were seeded on a CNT film and the cellular response was evaluated. The cells on the CNT film spread out evenly and displayed an elongated morphology by day four in culture. The presence of filopodia observed in the cells cultured on the CNT films confirmed the ability of the cells to spread better on CNT films than on the control. In addition, parallel entanglement of the actin filament network of the cells cultured on the CNT films was detected, which endorsed the favorable impact of CNTs on cell adhesion. The cellular adhesion of osteoblasts on CNT-coated collagen sponges was also concluded in a study by Li et al. [49].

Both GO and CNT have been provn to be good substrates for growth of different types of stem cells that promote cellular adhesion and well-developed actin filaments. Taken together, in our study, materials with GO and CNTs improved hASCs adhesion in a proportional manner to the GO-CNT concentration. Moreover, these carbon-based nanomaterials showed the potential to further influence the differentiation of stem cells towards osteogenic or adipogenic lineages [50].

Graphene and its derivates were shown to have the potential to induce differentiation of MSCs towards multiple lineages like osteogenic, adipogenic, and chondrogenic [13]. GO, in particular, based on its property to adsorb certain proteins, can accelerate adipogenesis of MSCs. Such a protein is insulin, essential for the synthesis of fatty acids, which can also be adsorbed on GO without altering its 3D conformation [4]. The gene and protein expression levels of adipogenic markers were found to be significantly higher in tonsil-derived MSCs when seeded on a hydrogel enriched with GO than on a hydrogel without GO [12]. Our results confirmed that the addition of 1 wt% GO and CNTs in a material’s composition enhanced the expression of adipogenic markers on both gene and protein levels in hASCs. Peripilin, as an adipogenic marker, displayed an increasing profile associated with higher concentrations of GO and CNTs, indicating their positive influence on adipogenesis evolution.

Several studies confirmed the potential of GO to promote osteogenesis [8,13,51]. Liu et al. [51] reported GO’s efficiency in promoting osteogenic differentiation of preosteoblasts from MC3T3-E1 cell line cultured on GO-gelatin composite. The same conclusion was drawn seeding hASCs on GO-based substrates in a study by Suhito et al. [13] that showed enhanced expression of osteogenic markers indicating a more advanced osteogenic differentiation in the presence of GO. In one of our previous studies [52], hASCs were induced to differentiate on osteogenic lineage inside GO-chitosan scaffolds for 28 days. The hASCs were able to undergo osteogenesis and express specific markers with higher levels correlating with a greater concentration of GO in the scaffold. Our study complements these results and shows that along with the increase of the GO-CNT content in the CA-CNT-GO material, the expression of the bone development marker, osteopontin, was also amplified confirming GO’s osteoinductive potential.

CNTs promote better cellular spreading, which in turn influences the level of cell differentiation in a positive way through their property of mechanotransduction. CNTs are good inducers of cell differentiation of many lineages, among them chondrogenesis, neurogenesis, and osteogenesis [48]. CNT substrates have the potential to improve the differentiation of neural stem cells towards astroglial and neural cells and the differentiation of embryonic stem cells and mesenchymal stem cells [11]. In addition, it has been confirmed that CNTs can support adipogenesis as well [53], however, the studies that evaluate the adipogenic differentiation potential on CNT-based materials are limited. Our study contributes to this field by showing and confirming that adipogenic differentiation of hASCs was improved in contact with CA-CNT-GO membranes proportional to the GO-CNT concentration.

The improved osteogenic differentiation on CNT films is considered to be induced by the roughness of the material that stimulates the cells to spread more and the actin filaments to be reinforced [49,54]. Many studies support the positive influence of CNTs on the progress of osteogenesis. In one of these studies conducted by Zanello [55], the cells produced hydroxyapatite in the same way as cultured on woven bone. In addition, it was proposed that CNTs’ affinity for calcium is one of the reasons they promote osteogenic cell differentiation so well [54,56]. The positive role of CNTs on osteogenesis was underlined in our study as well, as the osteogenic differentiation was enhanced on CA-CNT-GO films as compared with the CA control.

Taking the properties of GO and CNTs together, the biomaterials we tested showed improved adipogenic and osteogenic differentiation over the 21 days of experiment, and a higher level of specific markers were associated with higher concentrations of GO and CNTs in the material’s composition.

## 4. Materials and Methods

Ammonia functionalized graphene oxide, cellulose acetate with Mn ~30000, 2-propanol (reagent Ph.Eur, ≥99.8%,) and N,N’-Dimethylformamide (DMF), 99% were acquired from Sigma Aldrich, USA. Ammonia functionalized short double-walled carbon nanotubes (DWCNT, 90+ C purity& NH2, 5% superficial functionalization) were procured from Nanocyl Belgium. As nonsolvent and for materials purification distilled water was used.

All materials for the differentiation studies were purchased from Thermo Fisher Scientific (Grand Island, NY, USA), unless otherwise stated. Cell media and supplements were purchased from Sigma-Aldrich (Steinheim, Germany) and ThermoScientific.

### 4.1. CA-CN-GO Membrane Synthesis and Characterization by Micro-CT

Cellulose acetate solution with 12 wt% concentration was obtained by dissolving the polymer in N,N’-dimethylformamide under constant stirring. A total 0.5 g of sodium hydroxide was added per 100 mL of solvent in order to increase the amount of accessible hydroxyl groups at the surface of polymer chains and to expand the compatibility between GO and CNTs with polymer. CNTs and GO powder (1:1 *w*/*w*) corresponding to 0.25/0.5/1 wt% of the polymer mass were added and 1.5 h ultrasound treatment was applied for nanofillers dispersion and achievement of homogenous solutions. Three cellulose acetate composite formulations were obtained: CA-CNT-GO 0.25%, CA-CNT-GO 0.5%, and CA-CNT-GO 1%, whereby the percentage stands for the total percentage of GO and CNTs. The solution was set under constant stirring overnight then poured in portions in a rectangular container, carefully levelled with a glass slide (doctor’s blade method [57]) and abruptly immersed in a coagulation bath consisting of 2-propanol distilled water in a *v*/*v* ratio of 1:3. Levelling was performed to remove the excess of solution and to keep the thickness of the membrane constant. After precipitation, the nanocomposite materials were left for a few hours to purify in distilled water and stored in plastic containers, in distilled water.

Materials were synthesized as previously described [41] and exposed to ultrasound treatment. A VCX750 ultrasonic processor for small and medium volume applications (Sonics & Materials, Inc. Newton, CT, USA), with a probe tip manufactured of Ti-6Al-4V and the source operating with 750 W and 20 kHz was used for ultrasound treatment. The amplitude was set to 100% and a short-pulse sonication was done in 3 cycles of 30 min sonication and 15 min breaks. In order to avoid materials alteration during the ultrasound an ice bath container was used.

For micro-CT analysis, one specimen of each membrane (approximately 1 × 3 mm) was analyzed with a Bruker microCT 1172 high-resolution micro-computer tomography scanner without further treatment. The 4 samples were scanned with no filter, at a source voltage of 50 kV and a current intensity set for 200 µA, respectively, and an exposure per frame of 300 ms. Overall, the scanned datasets were acquired during 180° rotations of the sample, with a rotation step of 0.1° and frame exposure of 1200 ms. Each slice was averaged from 3 successive acquisitions. Image pixel size, explicitly the minimum resolution of the scanned dataset, was fixed at 0.5 µm for all specimens. In order to lessen the miscalculations, porosity analysis was not performed on the entire volume of the membrane. Raw data reconstructions were performed in Bruker NRecon software (version 1.7.1.6, Bruker, Billerica, MA, USA). Reconstructed tomograms were depicted in Bruker CTVox (version 3.3.0r1403 (64-bit), Bruker, Billerica, MA, USA), while quantitative determinations were performed using Bruker CTAn software (version 1.18.4.0+ (64-bit), Bruker, Billerica, MA, USA). For the three composite membranes and the control, 4 rectangular volume of interest (VOI) datasets were extracted. The VOIs were constrained in terms of volume and height (400 slices). In CTAn, the VOIs were processed through a common routine individualized for each specimen, consisting of thresholding to clear-cut the sample walls from the pores, despeckling for the removal of residual scanning artefacts, and 3D analysis to quantify specific surface, total porosity, structure separation, and structure thickness. No other image processing technique was suitable for the investigation.

Both pore size distribution and pore shape were altered as a result of the addition of GO and CNTs. This could be attributed to the gradual increase in hydrophobicity of the mixture upon the addition of GO and CNTs. Hence, the affinity of the casting composite solution mixture to the nonsolvent weakened. Upon nonsolvent immersion, hydrophobe filler started to segregate towards the membrane edges and initiate a premature demixing surge that also engaged the CA network. The tendency to generate larger pores within the membrane was in direct correlation with the nanofiller content. We assumed that 0.5 wt% GO-CNT concentration was above a threshold value whereby the repulsion kinetics of the hydrophobe nanomaterial and nonsolvent could generate enough energy to engage large volumes of the polymer along. After contact with water, GO and CNTs (when in higher amount 0.5 and 1 wt%) started to thrust from within the matrix and congest the medial domain bilaterally, generating additional partition through the medial lamina. This phenomenon was facilitated by the large interfacial contact area between the GO and CNTs with the enveloping polymer, on the grounds of the immense surface area of the nanomaterial, and the excellent dispersion efficiency.

### 4.2. Achievement of Cell-Material Systems

Human adipose-derived stem cells were used for both cell adhesion and differentiation experiments. Cells were obtained from liposuction after patient informed consent by using a well-established protocol [58,59]. All cell-based experiments were approved by the Ethics Committee of the University of Bucharest and were in compliance with the Declaration of Helsinki. The cells were grown in Dulbecco’s Modified Eagle’s Medium (DMEM, Sigma-Aldrich, Darmstadt, DE) supplemented with 10% FBS and 1% antibiotic-antimycotic (Sigma-Aldrich, Darmstadt, DE) at 37 °C in a 5% CO_2_ atmosphere. The medium was changed once every 3–4 days.

Materials were prepared and seeded with hASCs at 2 × 10^4^ cells/cm^2^ and the resulting bioconstructs were maintained in standard culturing conditions (37 °C in a humidified atmosphere containing 5% CO_2_) in complete medium for 24 h.

### 4.3. Adhesion of hASCs on CA-CNT-GO Films

After 24 h of culture, adhered cells on the films were fixed with a 4% paraformaldehyde solution (Sigma-Aldrich, Darmstadt, DE) for 20 min, permeabilized with 2% BSA solution with 0.1% Triton X100 (Sigma-Aldrich, Darmstadt, DE) for 1 h, and stained with fluorescein-conjugated phalloidin (Sigma-Aldrich, Darmstadt, DE) for 20 min and 4, 6-diamidino-2-phenylindole (DAPI, Sigma-Aldrich, Darmstadt, DE) for 5 min. The cells were visualized by fluorescence microscopy (IX-73 Olympus). The quantification of phalloidin-FITC, was made with Image J software and plots were obtained with GraphPad Prism version 6 for Windows.

### 4.4. Differentiation Induction in hASCs CA-CN-GO Systems

After 24 h of cell culture, the complete medium was changed to a commercially available cocktail of adipogenic inducers (StemProAdipogenesis Differentiation Kit, Thermo Fischer Scientific, Waltham, MA, USA) or osteogenic inducers (StemPro Osteogenesis Differentiation Kit, Thermo Fischer Scientific, Waltham, MA, USA). The differentiation process was monitored for 21 days. The medium was changed every 3 days. The expression levels of adipogenic- and osteogenic-specific markers, perilipin and osteopontin, were evaluated after 7, 14, and 21 days, respectively. The accumulation of intracellular lipids was highlighted by Oil Red O (ORO) staining at 7 and 21 days, while the mineralization of the extracellular matrix (ECM) was evaluated on days 14 and 21 by Alizarin Red S (ARS) staining.

### 4.5. Gene Expression Analysis of Specific Differentiation Markers by qPCR

Total RNA was collected using TRIzol (Thermo Fisher Scientific, Waltham, MA, USA), RNA integrity number (RIN) was determined using Agilent 2100 bioanalyzer and cDNA was synthesized using an iScript cDNA synthesis kit (BioRad, Hercules, CA, USA). cDNA was amplified by PCR using Veriti 96-Well Thermal Cycler from Applied Biosystems. Real-time PCR was performed using FastStart DNA Master SYBR Green I kit and LightCycler 2.0 from Roche. Every sample was evaluated in triplicate, and the expression of TATA-binding protein (TBP) was used as a reference. The primers used were: tbp sense 5′ AGGCATCTGTCTTTGCACAC 3′, tbp antisense 5′ GGGTCAGTCCAGTGCCATAA 3′, perilipin sense 5′ ATGCTTCCAGAAGACCTACA 3′, perilipin antisense 5′ CAGCTCAGAAGCAATCTTTT 3′, osteopontin sense 5′ GAGGAGGCAGACACAGCATC 3′, osteopontin antisense 5′ GCTTCTGAGATGGGTCAGGG 3′.

### 4.6. Immunofluorescence Staining and Fluorescence Microscopy of Specific Differentiation Marker Proteins

Samples were fixed with a 4% paraformaldehyde solution for 20 min, permeabilized with 2% BSA solution with 0.1% Triton X100 for 1 h. The samples were then incubated with osteopontin antibody (for osteogenic differentiation) or perilipin antibody (for adipogenic differentiation) for 12 h at 40 °C, after which they were incubated with anti-mouse FITC conjugated secondary antibody (SantaCruz, Heidelberg, Germany) for 1 h. Cell nuclei were stained with DAPI solution (Thermo Fisher Scientific, Waltham, MA, USA) and the cells were visualized by fluorescence microscopy (IX-73 Olympus). The quantification of perilipin and *OPN* levels was made with Image J software and plots were obtained with GraphPad Prism version 6 for Windows.

### 4.7. Histological Stainings to Prove Differentiation Evolution

Oil Red O (ORO) staining was used to highlight the amount of neutral lipids accumulated during adipogenic differentiation. Cells were fixed in 4% paraformaldehyde (Sigma-Aldrich, Darmstadt, DE) at 4 °C, permeabilized with 2% BSA solution with 0.1% Triton X100 for 1 h and stained with 5 mg/mL Oil Red O solution for 2 h at room temperature. The lipid droplets of the cells were observed at 7 and 21 days after the induction of adipogenesis.

Alizarin Red S (ARS) staining was performed to highlight the degree of mineralization of ECM during in vitro osteogenesis. For the ARS staining, the cells on the materials were fixed in 4% paraformaldehyde (Sigma-Aldrich, Darmstadt, DE), washed with PBS and stained with 2% ARS staining solution. The mineral deposits stained in bright red were observed under the microscope on days 14 and 21 after the induction of osteogenesis.

The images were acquired by IX-73 Olympus microscope. The quantification of ARS and ORO stained elements was made with Image J software and plots were obtained with GraphPad Prism version 6 for Windows.

### 4.8. Statistical Analysis

GraphPad Prism (version 6, San Diego, CA, USA) for Windows was used to statistically analyze results. All quantitative results are presented as mean ± standard deviation (SD) of *n* = 3 experiments and they were compared using one-way ANOVA and Bonferroni post-test. *p*-values < 0.05 were considered to be statistically significant.

## 5. Conclusions

In this study, we tested, for the first time, bidimensional biomaterials based on CA enriched with a combination of 0.25–1 wt% GO and CNTs for their ability to support interaction with cellular components and for their versatility as substitutes in different tissue engineering applications. The purpose of this research was to prove that a fine control of the application can be provided by modulating the combination of two carbon derivatives, CNTs and GO, in order to obtain the appropriate porosity and parameters for reconstruction of either soft or hard tissues. The CA-CNT-GO membranes displayed porous morphologies with open and interconnected voids which are believed to facilitate cell migration within the material. Overall, the porosity of the material slightly decreased as the nanofiller amount increased, resulting in the formation of slightly more compact materials (less pores but slightly more uniform dimensionally). A key parameter was the change of the morphostructural features as the amount of GO and CNTs increased to 0.5 and 1 wt%, i.e., the midway horizontal wall parallel to the peripheral surfaces and the decrease of thickness of exterior walls. The two events are foreseen to better facilitate cell migration and to promote the cell differentiation possibly more efficiently towards hard tissue due to higher mechanical support. During adipogenic differentiation, a higher number of cells was engaged in differentiation over 21 days, forming larger lipid accumulations, while the extracellular matrix produced during osteoblast development was found to be augmented proportionally to the GO-CNT concentration. Indeed, gene and protein profiling of both adipogenic and osteogenic specific markers confirmed the ability of these CA-CNT-GO substrates to support both types of soft and hard tissue engineering, thus their versatility for biomedical applications.
